# How Water Binds to Microcline Feldspar (001)

**DOI:** 10.1021/acs.jpclett.3c03235

**Published:** 2023-12-29

**Authors:** Giada Franceschi, Andrea Conti, Luca Lezuo, Rainer Abart, Florian Mittendorfer, Michael Schmid, Ulrike Diebold

**Affiliations:** † Institute of Applied Physics, TU Wien, 1040 Vienna, Austria; ‡ Department of Lithospheric Research, Universität Wien, 1090 Vienna, Austria

## Abstract

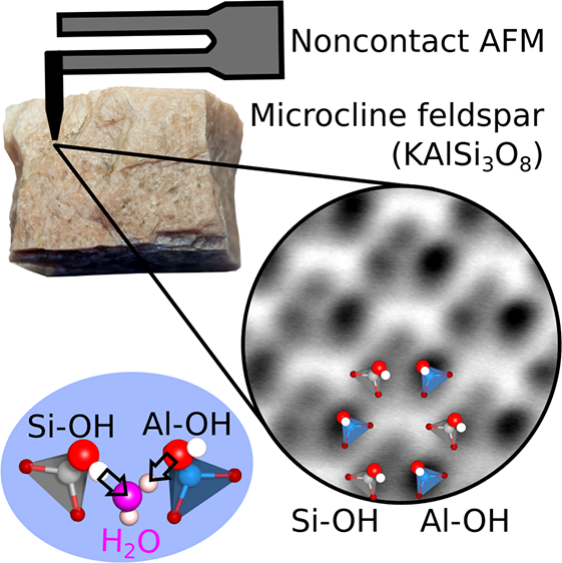

Microcline feldspar (KAlSi_3_O_8_) is a common mineral with important roles in Earth’s ecological balance. It participates in carbon, potassium, and water cycles, contributing to CO_2_ sequestration, soil formation, and atmospheric ice nucleation. To understand the fundamentals of these processes, it is essential to establish microcline’s surface atomic structure and its interaction with the omnipresent water molecules. This work presents atomic-scale results on microcline’s lowest-energy surface and its interaction with water, combining ultrahigh vacuum investigations by noncontact atomic force microscopy and X-ray photoelectron spectroscopy with density functional theory calculations. An ordered array of hydroxyls bonded to silicon or aluminum readily forms on the cleaved surface at room temperature. The distinct proton affinities of these hydroxyls influence the arrangement and orientation of the first water molecules binding to the surface, holding potential implications for the subsequent condensation of water.

Feldspars are tectosilicates made of corner-sharing AlO_4_ and SiO_4_ tetrahedra and varying ratios of Ca, Na, and K ions. They are ubiquitous and participate in maintaining our planet’s delicate equilibrium. Feldspars largely compose the rocks we stand on and are active at sequestrating atmospheric CO_2_.^1^ Through weathering processes, they transform into clays and create soils, providing essential nutrients for plant growth.^2^ Furthermore, they exist as airborne dust particles in the atmosphere, where they influence ice nucleation (IN) and cloud formation, profoundly impacting global weather patterns.^3^ While all these crucial processes occur on the surfaces of feldspars, the current knowledge about the atomic structure of feldspar surfaces, and how it may affect their interaction with the environment, stems largely from computational works. Experimentally, most information regarding surface processes of feldspars is inferred from either indirect or bulk measurements.

The lack of detailed knowledge of the surface chemistry of feldspars is evident in current research on ice nucleation. K-feldspars (KAlSi_3_O_8_) and particularly the lowest-temperature polymorph known as microcline (Figure 1) are exceptionally active ice-nucleating agents in the atmosphere.^4−10^ Many theoretical studies have tried to correlate surface chemistry and IN activity by investigating the atomic-scale interaction of “perfect” microcline surfaces with water. Ab initio DFT calculations have shown that ice-like structures can grow atop a non-ice-like, mediating water layer directly adsorbed on the lowest-energy (001) surface of microcline.^11^ However, molecular dynamics studies have fallen short in replicating spontaneous IN on microcline’s low-index facets, even at temperatures well below the freezing point of water.^12,13^ On the experimental front, studies on IN have predominantly relied on the observations of macroscopic ice crystals, focusing on the potential role of macroscopic defects on microcline rather than its surface chemistry.^9,14−17^ To bridge current theoretical and experimental studies, direct atomic-scale investigations of pristine microcline surfaces and their interaction with water are needed. Such studies may shed light on microcline’s ability to support hydrogen-bonded networks—an important factor for ice nucleation on other silicate minerals.^18^

**Figure 1:**
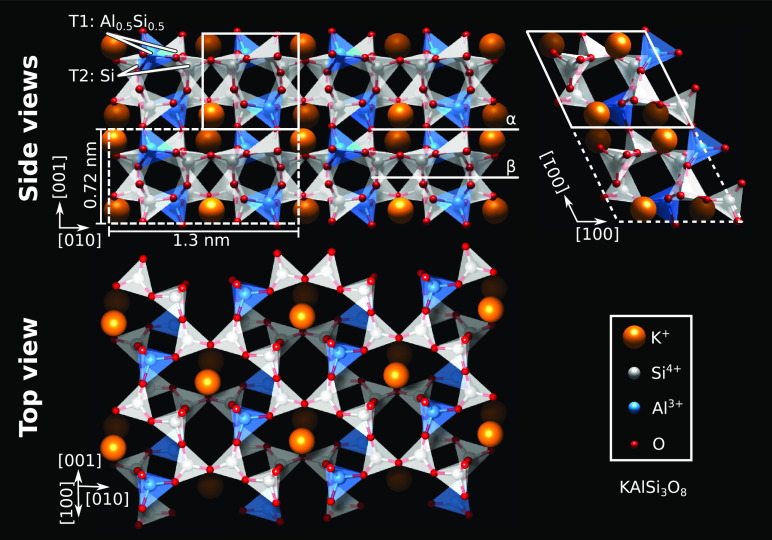
DFT-optimized bulk structure of microcline feldspar. Side and top views of the lowest-energy (001) plane. Polymerized AlO_4_^–^ and SiO_4_ tetrahedra form a 3D network in the “mirror-crankshaft chain” configuration. The primitive unit cell (white, solid) contains 2 AlO_4_^–^ tetrahedra (blue), 2 K^+^ ions (orange), and 6 SiO_4_ tetrahedra (gray). The conventional unit cell (white, dashed), also used for the DFT calculations, contains double the atoms. Al ions sit exclusively at T1 sites, as opposed to higher-temperature feldspars, where they can occupy both T1 and T2 sites. The α and β cuts explored in this work are marked.

Microcline’s crystal structure is shown in Figure 1. It is triclinic and centrosymmetric, comprising a 3D framework of corner-sharing SiO_4_ and AlO_4_ tetrahedra with large cavities housing K ions. Cleaving along the (001) plane occurs easily; stacking along this direction comprises layers of K, mixed SiO_4_ and AlO_4_ tetrahedra, and SiO_4_ tetrahedra. How microcline (001) is terminated after cleaving is debated.^13,19^ Candidate cleaving planes are denoted as α and β in Figure 1. Cleaving along plane α requires that half of the number of bonds are broken as compared to plane β and should hence be favored. However, when hydroxylation is considered, plane β becomes more stable.^13^ Note that the Al ions occupy only the T1 sites in the tetrahedral framework. Following Löwenstein’s rules for aluminosilicates, the Al ions that occupy 50% of the T1 sites will arrange in an ordered manner to minimize the overall electrostatic energy.^20^ This makes microcline a “well-ordered” feldspar, in contrast to higher-temperature polymorphs where the Al ions are distributed among the T1 and T2 sites.^21^

This work aims to unveil the atomic structure of the cleaved (001) surface of microcline feldspar and its interaction with water under controlled conditions. Direct experimental investigations by atomically resolved noncontact atomic force microscopy (AFM) with a qPlus sensor^22^ are complemented by X-ray photoelectron spectroscopy (XPS) and density functional theory (DFT) calculations. The measurements build on previous atomically resolved investigations of water structures adsorbed on ordered surfaces^23−30^ but take the significant leap forward of tackling a large band gap material such as microcline. Microcline (001) is found to cleave at plane α, which readily hydroxylates at 300 K even when cleaving in ultrahigh vacuum (UHV) because of water inclusions in the sample. The resulting surface hydroxyls (bonded to either Si or Al) are arranged in a buckled honeycomb pattern and template the adsorption of H_2_O molecules in an ordered fashion.

The (001)-oriented natural microcline mineral specimen used for the present UHV study was characterized ex situ by microprobe analysis and photomicrography using (001)-oriented thin sections (Figure S1). The sample is largely composed of microcline but also features small and sparse domains of Na-rich feldspar (albite), small quartz inclusions, and accessory hematite inclusions that give the mineral a reddish stain. Present are also submicrometer-sized inclusions of clay minerals, i.e., hydroxyl-bearing sheet silicates. The XPS data acquired on UHV-cleaved feldspar are in line with the ex situ characterization. The survey and K 2*p* + C 1*s* spectra in Figure S2 show that the cleaved surface is free of contaminants and features the expected elements (K, Si, Al, O), plus a minor contribution of Na, likely from the Na-rich feldspar regions.

As expected from its known cleaving properties, the (001) microcline surface appears flat in ambient AFM images (Figure 2a). Terraces are hundreds of nanometers in size and are separated by steps with heights that are multiples of the unit cell. Occasionally, areas with smaller terrace sizes were observed (Figure S3). The surface also appeared flat in AFM after UHV cleavage (Figure 2b). It is well-ordered (see the Fourier transform in the inset), except for sparse bright and dark point defects. The appearance of the defect-free areas depends sensitively on the tip termination (Figure S7) and relative tip–sample distance (Figure S8). The sharpest tips (either Cu or CuO_*x*_^31^) produce the contrast shown in Figure 2c: a distorted honeycomb lattice (black) framed by two sets of differently attractive features, plus an additional feature inside the honeycomb (asterisk).

**Figure 2:**
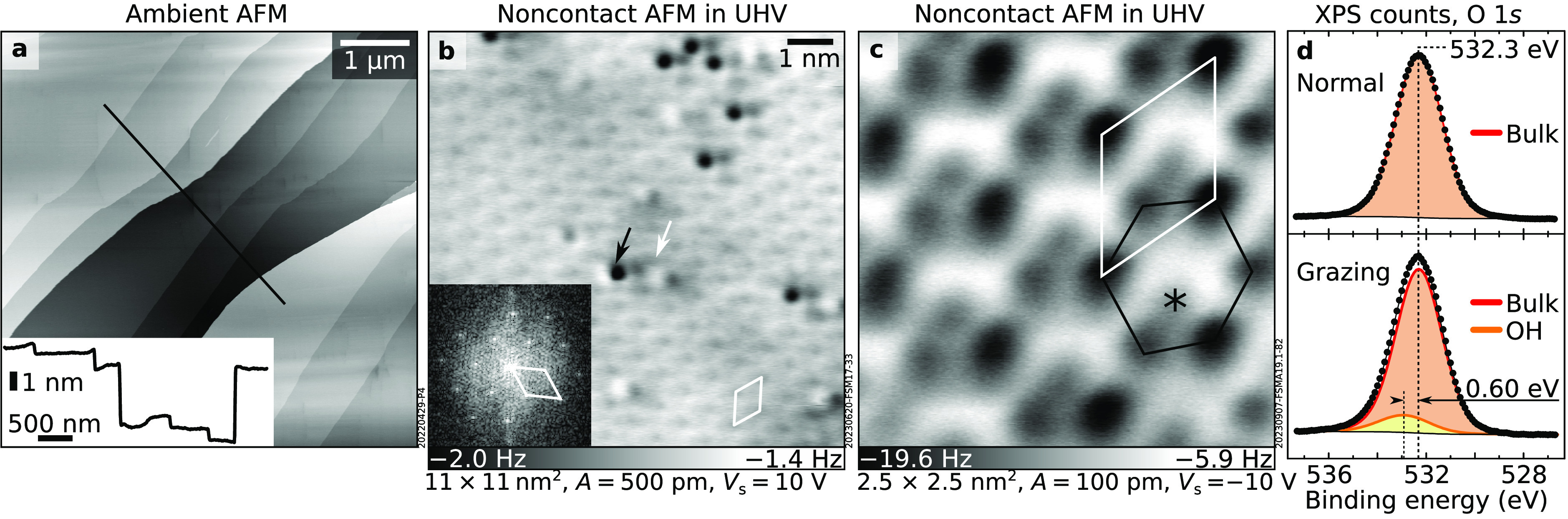
Cleaved microcline feldspar (001). (a) 6 × 6 μm^2^ ambient AFM image. Inset: line profile taken at the solid line in the main panel. (b, c) Constant-height, noncontact AFM images of the UHV-cleaved surface. (b) Overview acquired with a CuO_*x*_-terminated tip. Two types of point defects highlighted by arrows are visible on a regular lattice. Inset: Fourier transform of panel b. (c) Small-area image acquired with a Cu-terminated tip. Highlighted are the honeycomb lattice with two sets of differently protruding features (black), a feature inside the honeycomb (asterisk), and the primitive unit cell (white). (d) O 1*s* core-level peaks in normal and grazing emission normalized to the area of the respective bulk components. The grazing-emission spectrum can best be fitted by adding a small contribution (yellow) at a binding energy higher than that of the main peak.

During cleavage, a water pressure burst was observed in the UHV chamber. The water may be derived either from the clay inclusions in the microcline mineral grain or from micro- and nanometer-sized fluid inclusions that are typically associated with the interfaces between K-rich and Na-rich domains (see Section S2). While XPS cannot directly detect hydrogen, core-level shifts of elements to which H may bind, such as O, can be used to deduce the presence of water or hydroxyls on the surface. Figure 2d compares XPS O 1*s* peaks acquired on a UHV-cleaved surface in normal and grazing emission. The normal-emission peak, dominated by subsurface layers, is fit by one component (532.30 eV after binding energy correction for charging; see Section S1). The more surface-sensitive grazing-emission spectrum features a slightly shifted peak. Below, this is explained as an additional contribution at a higher binding energy due to surface OH groups forming when water is released during cleaving.

H_2_O vapor was dosed at 100 K on the cleaved surface, and the evolution of the surface was followed in both XPS and AFM (Figure 3). In grazing-emission XPS (Figure 3a), a third component grows in the O 1*s* region. It is separated by 1.2 eV from the main component and is associated with molecular H_2_O. In AFM (Figure 3b–d), dark (attractive) features appear on the surface that gradually fill up a hexagonal lattice with the same periodicity as the cleaved surface. The tip can interact with these features and displace them to different lattice positions (in Figure 3c, the circle highlights such an event; arrows indicate three water species displaced due to interaction with the tip). The attractive contrast in Figure 3b was obtained with a Cu-terminated tip. Figure 5c shows an image acquired with a CO-terminated tip, evidencing a bright (repulsive) contrast of the water species instead.

**Figure 3:**
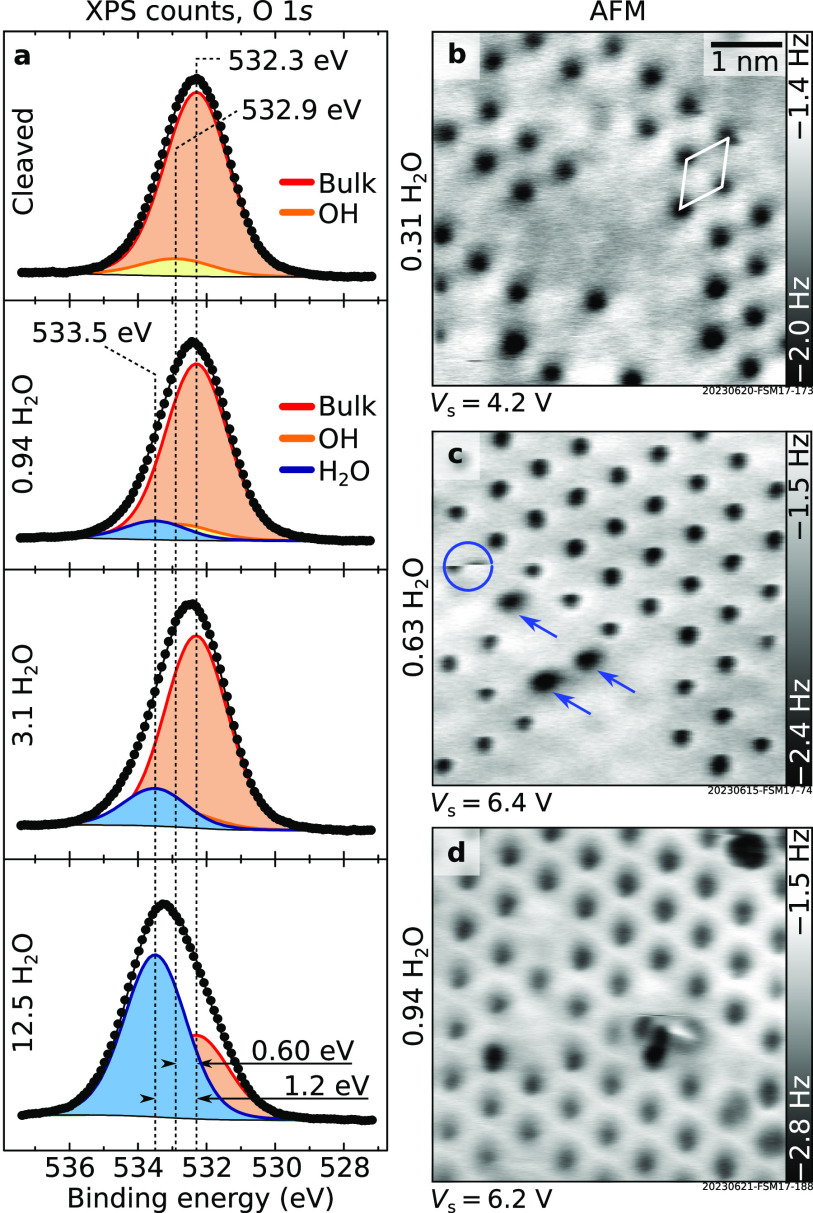
Adsorption of H_2_O at 100 K. (a) Experimental data (dots) and fits (solid lines) of the O 1*s* core-level peaks of the UHV-cleaved microcline exposed to H_2_O vapor at 100 K (Al Kα, pass energy 20 eV, 70° grazing emission; doses are expressed as the number of H_2_O molecules per primitive unit cell dosed at 100 K). (b–d) 6 × 6 nm^2^ constant-height AFM images of microcline (001) exposed to H_2_O vapor at 100 K. All images were acquired with a qPlus sensor and oscillation amplitude *A* = 500 pm; see Section S1 for *V*_s_. In panel b, the unit cell is highlighted in white. From one experiment to the next, the tip was slightly modified through an interaction with water species. In panel c, the circle highlights such an interaction event; arrows indicate three water species that have been displaced from their original lattice position due to the interaction with the tip.

If the sample dosed with H_2_O at 100 K is warmed to 300 K, the surface recovers the same appearance as an as-cleaved sample (Figure S10); that is, the features observed in Figure 3b–d desorb from the surface. A desorption temperature between 150 and 160 K was estimated from XPS (Section S1).

DFT calculations were performed for two different terminations of microcline (001), namely, the α (between K planes) and the β (between Si–Si planes) cuts (Figure 1). Calculations were performed on the dry as well as on water-exposed surfaces. The full set of calculations is discussed in detail in Section S3. Figure 4a–c focuses on the results obtained on the α cut. Based on the phase diagram in Figure S4a, plotting surface energies as a function of the water chemical potential, the α cut is the most stable termination in a wide range of experimental conditions: At a temperature of 300 K, it has the lowest energy across pressures ranging from UHV to ambient pressure.

**Figure 4:**
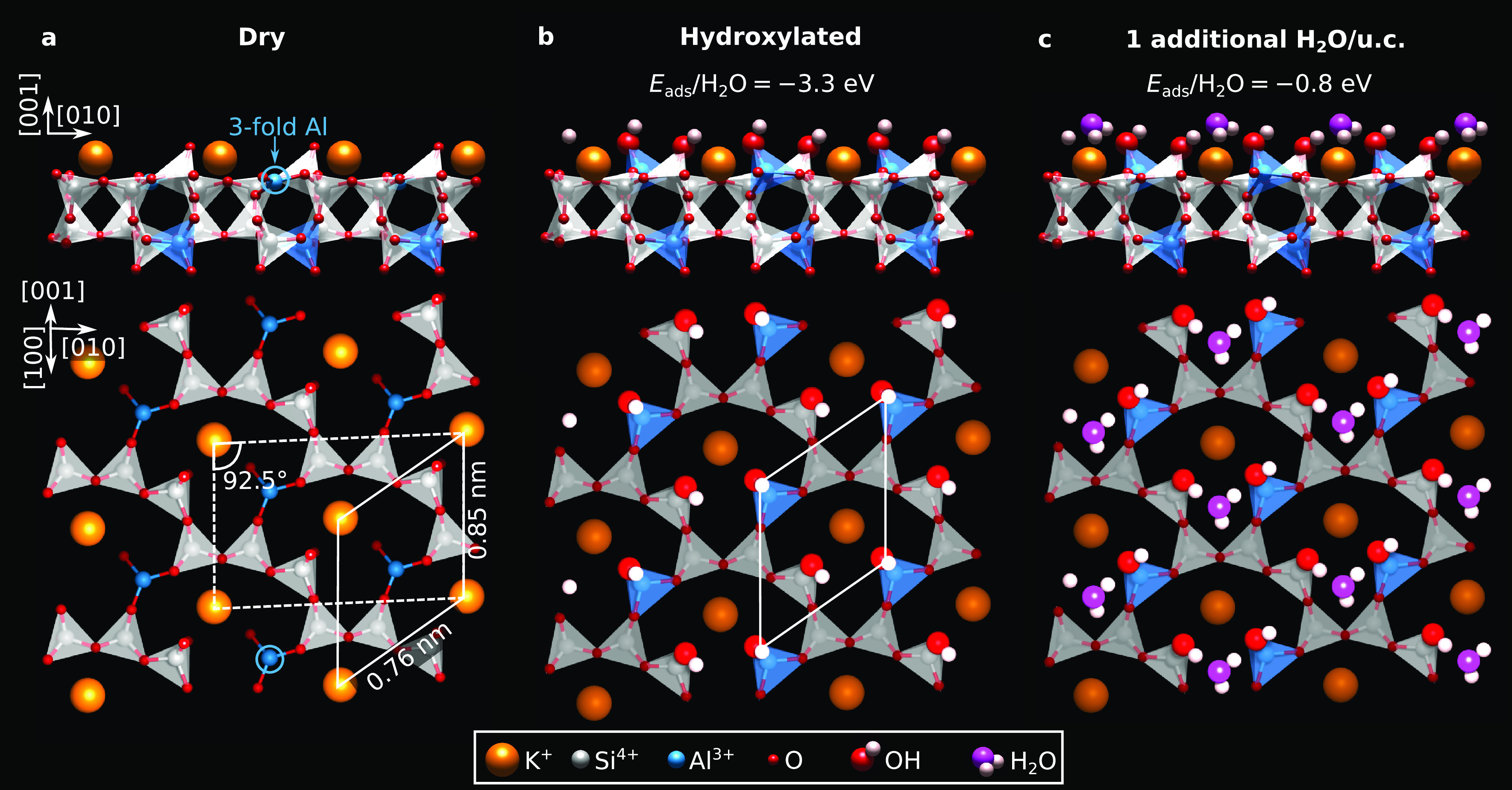
DFT-relaxed α cuts of microcline feldspar (001). (a) No water adsorbed. (b) One dissociated H_2_O/u.c. (c) One additional H_2_O molecule/u.c. over the hydroxylated surface. Relative adsorption energies are reported above the corresponding models. The conventional and primitive unit cells are marked with dashed and solid lines, respectively.

As seen from Figure 4a, the relaxed dry α cut is essentially bulk truncated. Cleaving breaks the surface O–Al bonds, leaving O atoms on the topmost Si atoms and producing undercoordinated surface Al. (Figure S5 shows that breaking O–Si or mixed O–Al and O–Si is less favorable.) Water readily dissociates on this termination (see Figure 4b), with an adsorption energy of −3.3 eV/H_2_O. The first H_2_O molecule per primitive surface unit cell (u.c.) splits without a barrier, donating one proton to the Si-backbonded surface O atom and the split-off OH to the undercoordinated Al ion (Figure 4b), i.e., creating a silanol and aluminol species. A coverage of one H_2_O molecule per unit cell is enough to fully hydroxylate the surface. The large adsorption energy explains why the surface remained protonated during molecular dynamics simulations with large quantities of water.^13^ To explore how additional water adsorbs on the fully hydroxylated α surface, calculations were run with one extra H_2_O molecule per u.c.. Consistent with computational results^11^ and the XPS data in Figure 3a, the additional molecule remains undissociated. As seen from Figure 4c, it accepts a hydrogen bond from Si–OH and donates one to Al–OH. In agreement with ref (11), the molecule adsorbs with ∼ −0.8 eV binding energy.

Figure 5 compares experimental AFM images of the cleaved and water-dosed surface with AFM simulations from the theoretical models of Figure 4b,c. Simulated images of the hydroxylated α cut reproduce the AFM contrast on the cleaved surface (see Figure 5a,b, obtained with CuO_*x*_ tips; Figure S8 shows results obtained with Cu-terminated tips instead). Both CuO_*x*_ (Figure 5a) and Cu tips (Figure 2c) show a honeycomb pattern. Each honeycomb is composed of two sets of species with different contrast, highlighted by the black and white circles in Figure 5a. The darker set (stronger attractive interaction of the AFM tip; marked by black circles) to the most protruding Al–OH, and the fainter (white circles) is assigned to Si–OH. A faint feature is observed inside the honeycomb and is marked by an asterisk (this is more evident with sharper tips, see Figure 2c). Based on the correspondence with the DFT relaxed model, it is assigned to the highest-lying K ion. Note that microcline has a centrosymmetric crystal structure. Hence, the (001) and  terminations should be mirror symmetric. Consistently, mirror-symmetric AFM simulations and experimental images are obtained on opposite terminations (Section S4).

**Figure 5:**
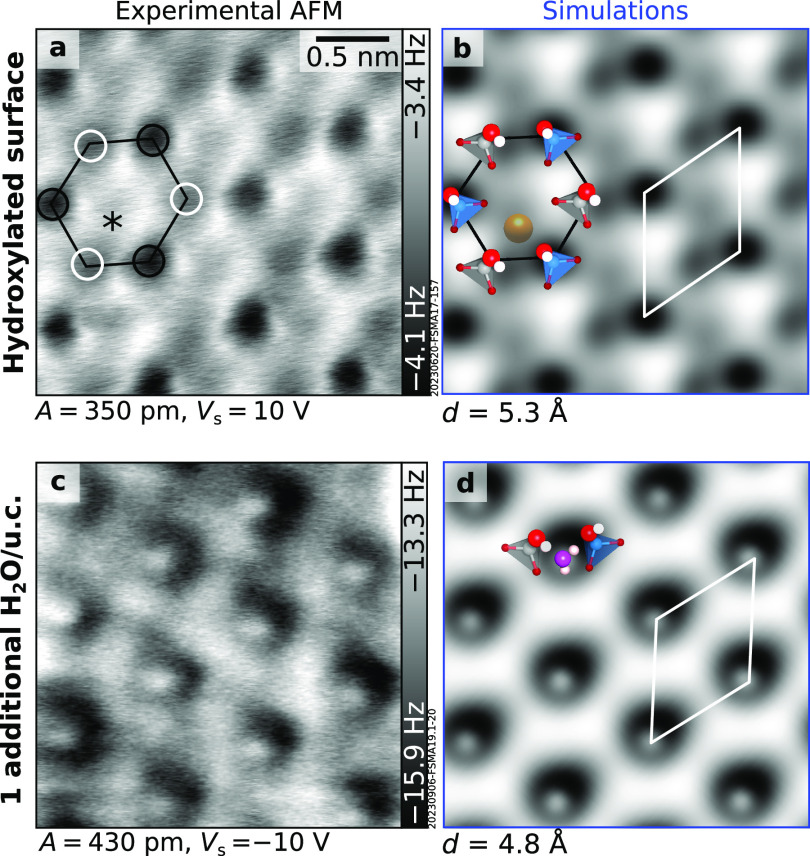
Comparison between experimental and simulated AFM images. Experimental (black frames, left) and simulated (blue frames, right) AFM images of the (a, b) hydroxylated microcline (001) α surface and (c, d) the same surface after dosing one additional water molecule per primitive unit cell (u.c.) at 100 K. All images are 2.5 × 2.5 nm^2^. White rhombi identify the primitive unit cell. In panel a, black and white circles mark hydroxyls bound to Al and Si, respectively. The asterisk marks the feature assigned to K. AFM simulations were performed at the tip–sample distances noted below the corresponding images. Different tips were used in both experiments and simulations: (a, b) CuO_*x*_-terminated; (c, d) CO-terminated. Figure S8 shows the experimental and simulated images of both surfaces with Cu tips.

A good match is also obtained between the experimental AFM image of the cleaved surface dosed with one H_2_O molecule per unit cell at 100 K (Figure 5c) and the simulation obtained from the model of Figure 4c, i.e., one additional H_2_O per unit cell on top of the hydroxylated α cut (Figure 5d). Both show a hexagonal pattern of protruding features with the same unit cell as that of the hydroxylated surface. These features are imaged in the repulsive regime (bright) with a CO-terminated tip and in the attractive regime (dark) with a Cu-terminated tip (Figure S8).

The computational data presented in Section S3 show that the α cut is more stable than the β cut under UHV conditions; that is, the sample should cleave between the K planes and relax to a quasi-bulk-truncated termination. If sufficient water is available in UHV, this termination should readily hydroxylate due to the large adsorption energy of H_2_O, as also evident from the phase diagram of Figure S4a. Previous literature reported that the hydroxylated β cut should be more stable than the hydroxylated α cut in UHV at 0 K.^13^ However, this situation cannot be obtained experimentally. The sample will cleave at the energetically preferred plane (the α cut, where the least number of bonds are broken). If enough water is available, then the α cut will become hydroxylated. Kinetics at room temperature is insufficient to switch to the hydroxylated β plane.

All evidence suggests that microcline (001) cleaves at the α cut and readily hydroxylates in UHV at 300 K, even without any intentional water supply. While one could consider that microcline cleaves preferentially at “special”, hydroxylated planes, the absence of step-bunching (see line profile in Figure 2a) speaks against this hypothesis. The water needed for hydroxylation is likely provided by clay or fluid inclusions in the natural minerals (see Section S2) exposed during the cleaving procedure. Based on the DFT-predicted adsorption energies, any available water molecules will stick with 100% probability on the microcline surface and dissociate without a barrier to form two hydroxyls. Based on mass-spectrometer measurements, the amount released through cleaving suffices for full hydroxylation (see Section S1 and Figure S10c). While it is somewhat surprising that the surfaces are immediately hydroxylated and “dry” surfaces are not produced even in the most pristine UHV environment, the energetics (see the phase diagram of Figure S4) suggest that the resulting, fully hydroxylated surfaces will also be present in ambient conditions.

The surface OH groups after cleavage are evidenced by a small component at higher binding energy in the grazing-emission O 1*s* spectrum (Figure 2b). This signal sits between the main O 1*s* component (532.30 eV) and the molecular H_2_O component obtained by dosing H_2_O at 100 K (533.5 eV, see Figure 3a), as typical for OH on other water-exposed oxides.^26,29,32^ The energy differences between the bulk O 1*s* peak and the ones assigned to OH and H_2_O peaks (0.60 and 1.20 eV, respectively) are reasonably reproduced by DFT calculations: Core-level-energy shifts of ∼0.5 eV and ∼0.9 eV are predicted for OH and H_2_O (details in Section S1). The presence of hydroxyls after cleaving is also supported by the identical appearance of the surface after dosing H_2_O at 100 K followed by warm-up to 300 K (Figure S10). Based on the strong adsorption energies of −3.3 eV predicted by DFT, the hydroxyls are expected to remain on the surface at 300 K. Finally, DFT predicts adsorption energies of −0.8 eV for H_2_O molecules adsorbed on the hydroxylated surface (Figure 4c); that is, temperatures lower than 300 K will be needed for adsorption. Consistently, XPS shows that molecular H_2_O starts desorbing between 150 and 160 K, corresponding to an adsorption energy of ∼−0.6 eV.^33^ The picture is validated by the good match between the experimental images and the simulations from the DFT-relaxed models (Figure 5).

The different types of hydroxyls found at the microcline surface (Al–OH and Si–OH) affect the anchoring of subsequent H_2_O molecules. The bond between Al and OH is weaker than the one between Si and OH due to the smaller charge of Al (3+) compared to Si (4+). As a result, the proton bound to Si–O should be released more easily than the one bound to Al–O; in other words, Si–OH should be more acidic than Al–OH. As seen from Figure 4c, such a difference in acidity influences the adsorption configuration of additional H_2_O molecules on the hydroxylated surface. As expected, the more acidic Si–OH donates an H bond to the H_2_O molecule, while the Al–OH accepts it. The model in Figure 4c is further supported by the good match between the experimental and simulated images seen in Figure 5.

That OH sites are important for stabilizing water molecules should not surprise. Under ambient conditions, hydroxyls exposed at oxide surfaces participate in the formation of wetting layers.^34,35^ At lower temperatures, the OH surface density is a good predictor of IN abilities.^36^ OH sites and the H bonds they offer appear to be more important than electrostatic interactions with surface K^+^ ions. The latter remain snug in their position, contrary to what happens upon immersion in liquid, where K ions are readily exchanged for protons.^19,37^ On the other hand, when there is no opportunity for surface OH groups, adsorption of an ordered array of H_2_O molecules may be challenging. Muscovite mica, another K-rich aluminosilicate of composition KAl_2_(Si_3_Al)O_10_(OH)_2_, exemplifies this. When cleaved in UHV, muscovite exposes undercoordinated K ions lying on an otherwise bulk-truncated surface.^38^ Water dosed at 100 K in UHV on this system adsorbs molecularly rather than dissociatively, completing the hydration shell of the surface cations and triggering the formation of 3D clusters rather than an ordered network of H_2_O molecules.^39^

It is interesting to compare the presented study to ice nucleation experiments performed on microcline crystals at real-world conditions. In the present work, XPS shows that H_2_O molecules dosed at 100 K desorb between 150 and 160 K in isobaric equilibrium measurements at a partial pressure of 1.5 × 10^–8^ mbar. This corresponds to a chemical potential of water  between −0.57 eV and −0.53 eV (see Section S1). These values are aligned with existing immersion-freezing^40^ and deposition-mode experiments^9^ on microcline, where ice condenses at  values between −0.54 eV and −0.55 eV. The matching values of the water chemical potential indicate that the conditions at which ice nucleates in the two cases are comparable. However, this alone is not enough to draw conclusions about the mechanism underlying ice nucleation on microcline. Macroscopic defects^9,14−17,41^ are known to play an important role for IN, but the circumstances leading to IN are not clear.^9^ A comparison of the IN activities of the same feldspar surfaces in immersion freezing and deposition modes showed that these provided two poorly correlated sets of active sites. A handful of sites though were active in both modes, pointing to a common nucleation mechanism.^9^ Interestingly, crystalline ice structures with the same epitaxial orientation were observed in both modes, a potential evidence that nucleation occurs on surface features of the crystalline substrate rather than on contaminants.^9^ The importance of the surface chemistry of crystalline phases is supported by the decreased IN efficiencies observed for amorphous silicates compared to crystalline ones.^42−44^ It is possible that the ordered anchoring of H_2_O molecules observed under UHV conditions offers the opportunity to create H-bonded water layers. In turn, this may relate to the observed crystalline ice structures. At this stage, however, it is premature to draw definite conclusions about the relative importance of surface chemistry versus surface defects for ice nucleation on microcline.

The specific superiority of microcline compared to other K-feldspars also remains up for debate. If one assumes that all K-feldspars have comparable macroscopic defects, the differences in their IN activities must relate to their intrinsic surface chemistry. By analogy with microcline, all (001)-oriented K-feldspars should cleave at the α cut and readily hydroxylate upon exposure to small quantities of water. The main difference between microcline and other polymorphs will be the number (smaller) and arrangement (more disordered) of the surface Al ions and, consequently, aluminol groups. As mentioned above, microcline is the most-ordered K-feldspar, with Al ions occupying only the surface T1 sites; in other feldspars, Al ions are distributed in surface T1 and subsurface T2 sites (see Figure 1). Since aluminol and silanol groups have different binding strengths and proton affinities,^11^ additional H_2_O molecules landing on disordered K-feldspars will find inequivalent, disordered, binding sites. This might disrupt the creation of an ordered first H_2_O adlayer, muddling the adsorption of additional water and decreasing the overall IN abilities.

In summary, this study combines UHV analyses by AFM and XPS with DFT calculations to investigate the atomic-scale details of microcline feldspar (001) and its interaction with water. The UHV-cleaved surface strongly reacts with water at room temperature, producing Si- and Al-bonded hydroxyls visible as a buckled honeycomb pattern in the atomically resolved AFM images. The different acidity of the long-range-ordered aluminol and silanol groups enforces a specific adsorption configuration for H_2_O molecules on this surface, carrying potential implications for the subsequent condensation of water molecules.

## Supporting Information Available

Section S1: Methods (UHV setup and characterization, ex situ characterization, computational details); Section S2: Further characterization of microcline feldspar (thin-section characterization, XPS, cleaving procedures, further ambient AFM images, optical approach in UHV); Section S3: Additional computational results (additional details about the β cut, additional details about the α cut, performance of r^2^SCAN and r^2^SCAN-D3 functionals compared, phase diagram as a function of the water chemical potential); Section S4: Considerations about symmetry; Section S5: Additional experiments and simulated images; Section S6: Δ*f*–*z* curves; Section S7: Arguments for the ready hydroxylation of the as-cleaved surface; Section S8: Imaging in the presence of surface charges (PDF)

## Abbreviations

IN: ice nucleation
AFM: atomic force microscopy
XPS: X-ray photoelectron spectroscopy
DFT: density functional theory
UHV: ultrahigh vacuum
