# Supplementary material for: How Water Binds to Microcline Feldspar (001)
Source: J Phys Chem Lett. 2023 Dec 29;15(1):15–22. doi: 10.1021/acs.jpclett.3c03235 (PMC10788961; doi:10.1021/acs.jpclett.3c03235)
Supplement: Supplementary file 1 — jz3c03235_si_001.pdf [file jz3c03235_si_001.pdf]

Supplementary Information for

How Water Binds to Microcline Feldspar (001)

Giada Franceschi,*[1] Andrea Conti,[1] Luca Lezuo,[1] Rainer Abart,[2] Florian Mittendorfer,[1] Michael Schmid,[1] Ulrike Diebold[1]

[1]Institute of Applied Physics, TU Wien, 1040 Vienna, Austria
[2]Department of Lithospheric Research, Universität Wien, 1090 Vienna, Austria

*Corresponding author. Email: franceschi@iap.tuwien.ac.at

This file includes:

Section S1: Methods
- UHV setup and characterization
- Ex-situ characterization
- Computational details

Section S2: Further characterization of microcline feldspar
- Thin-section characterization
- XPS
- Cleaving procedures, further ambient AFM images, optical approach in UHV

Section S3: Additional computational results
- Additional details about the β cut
- Additional details about the α cut
- Performance of r^2SCAN and r^2SCAN-D3 functionals compared
- Phase diagram as a function of the water chemical potential

Section S4: Considerations about symmetry
Section S5: Additional experimental and simulated images
Section S6: Δ*f*-*z* curves
Section S7: Arguments for the ready hydroxylation of the as-cleaved surface
Section S8: Imaging in the presence of surface charges

Supplementary References

Section S1: Methods

UHV setup and characterization

The experiments were carried out in a UHV setup consisting of two interconnected chambers: A preparation chamber for sample cleaving and XPS measurements and an adjacent chamber for AFM measurements (base pressures below 1×10^{-10} mbar and 2×10^{-11} mbar, respectively).

Natural microcline feldspar from Russia obtained by kind concession of Prof. Uwe Kolitsch from the Natural Museum of History of Vienna was characterized ex situ before the UHV measurements (see Section S2, reporting results from photomicrography, electron probe microanalyzer, and ambient AFM). The samples were glued on Omicron-style stainless steel sample plates with UHV-compatible epoxy glue (EPO-TEK T7110-38). They were cleaved in UHV at room temperature before each experiment with one of the two methods shown in Figs. S3a, b, which yielded consistent results: (i) by using a wobble stick to apply a tangential force to a metal stud glued on top of the sample.[1] The sample portion initially covered by the stud is thus cleaved and ready for XPS and AFM investigations; (ii) by using a guillotine-type cleaver[2] that shaved off the top part of the sample.

To study water adsorption on the microcline surface at low temperatures, water vapor was dosed from a leak valve while keeping the sample holder on the manipulator of the preparation chamber at 100 K with liquid nitrogen. The amount of water dosed on the surface is always expressed as the number of H_2O molecules per primitive unit cell (u.c.). The calibration was performed based on the dosing (water partial pressures × time) needed to obtain a coverage of 1 H_2O/u.c. at 100 K (assuming 100% sticking probability). This corresponds to 32 s at 1.3×10^{-8} mbar, in turn equal to 0.32 Langmuir (L, where 1 L is defined by an exposure time of 1 s at 1.3×10^{-6} mbar).

With each cleave, the partial pressure of water in the UHV chamber increased to $\approx 2 \times 10^{-7}$ mbar for a few seconds, for a total dose of ≈24 Langmuir (see Fig. S10c and related discussion). Since the hydroxylated surface requires a nominal dose of 0.32 L (the hydroxylated surface has the same density of H_2O molecules as the water-dosed surface), the observed 'pressure burst' should be enough to hydroxylate the surface fully.

XPS was performed with a non-monochromatic dual-anode Mg/Al X-ray source (SPECS XR 50) and a hemispherical analyzer (SPECS Phoibos 100). Spectra were acquired in normal and grazing emission (70° from the surface normal). The intensities and positions of the Al-Kα-excited XPS peaks were evaluated with CasaXPS after subtracting a Shirley-type background. Due to the insulating nature of the samples (bandgap of 7.6−7.7 eV, Ref. 3), the XPS spectra showed shifts to apparent higher binding energies (by 5−7 eV). The magnitude of the shift depends on the amount and type of surface adsorbates, XPS acquisition geometry, and sample thickness. For the display and analysis of the XPS data, an energy correction was applied to all spectra: The Si 2*p* core-level peak was set to 103.10 eV, as reported in the literature.[4] This resulted in an O 1*s* peak at a binding energy of 532.30 eV at normal emission.

Table 1 summarizes the constraints applied to the fits. The O 1*s* peak of the cleaved surface was fit by comparing normal and grazing emission acquisitions. The normal-emission spectrum

was fit by component 1 alone. Fitting the grazing-emission spectrum additionally required component 2. In the main text, component 2 is assigned to surface OH species that saturate the cleaved surface at room temperature. Increasing amounts of H_2O at 100 K induced the growth of a third component (assigned to molecular H_2O, see main text). Its position and FWHM were determined from high-dose experiments (>4 L, or 12.5 H_2O/u.c.), which were then constrained to fit the lower doses. For the fits of the low-temperature water experiments, the intensity ratio of components 1 and 2 was constrained to the value found on the cleaved surface, under the assumption that molecular H_2O grows on the fully hydroxylated surface (see main text).

Table 1. Details about the XPS fitting components of Fig. 2. The shape (LA=asymmetric Lorentzian), full-width half maximum, and position were constrained for all peaks.

	Identifier	Shape	FWHM	Position (eV)	Area
O 1*s* 1	O 1*s* cleaved	LA(1.53,243)	2.24	532.30	Free
O 1*s* 2	OH cleaved	LA(1.53,243)	2.5	(O 1*s* 1) + 0.60	(Area O 1*s* 1) × 0.117 (for molecular H_2O dosing)
O 1*s* 3	H_2O	LA(1.43,243)	2.2	(O 1*s* 1) + 1.20	Free

XPS was used to obtain an approximate isobar for molecular H_2O. Here 2 L H_2O were dosed at 100 K. In a water background pressure of ≈ 1.5×10^{-8} mbar, the sample was warmed to increasingly higher temperatures in steps of 10 K. XPS spectra (O 1*s*, K 2*p*, and Si 2*p* for energy correction) were acquired at each stage. To reach a situation as close to thermodynamic equilibrium as possible, the sample was kept in the water background for ≈ 20 min at each temperature before each measurement. The temperature where the coverage of the molecular water roughly halved was between 150 K and 160 K.

The AFM measurements were performed at 4.7 K using a commercial Omicron qPlus LT head and a differential cryogenic amplifier.[5] Frequency-modulated non-contact AFM mode was used. The tuning-fork-based AFM sensors (k = 2,000−3,500 N/m, $f_0 \approx 32$ kHz, Q ≈ 50,000) had a separate contact for tunneling current. The electrochemically etched W tips were cleaned by field emission.[6] Before each measurement, the tips were prepared on an oxygen-exposed Cu(110) single crystal by repeated indentation and voltage pulses. Cu-, CuO_x-, and CO-terminated tips were prepared on the oxygen-induced reconstruction of Cu(110)[7] to exhibit a frequency shift smaller (in absolute value) than −1.5 Hz. They were used to image the cleaved and water-exposed microcline surface. At times, the tip interacted with point defects (strongly attractive features, likely adsorbates) present on the surface; they easily snapped to the tip. The tip-to-sample approach was performed carefully to avoid the risk of crashing into the insulating, possibly charged, microcline surface. First, the tip was manually brought closer to a flat area of the surface, as judged by looking from an optical camera (Fig. S3d). This was followed by an automatic approach with a setpoint of −800 mHz, which ensures that the approach is stopped well before reaching the surface. Then, the controller was switched off and the tip was gradually approached in constant-height mode until an

AFM contrast was visible while scanning in x and y, at which point the tilt of the surface was corrected for. All the AFM images presented here were acquired in constant-height mode.

Like other insulators, the as-cleaved samples exhibit surface charges that make AFM measurements difficult.[8,9] The charge could be effectively remediated by irradiating the cleaved surface with X-rays from our XPS setup for one minute. This treatment did not introduce any spurious contaminations. Residual fields can be compensated by applying a bias voltage between the tip and sample. All measurements were performed by applying a bias voltage V_s to minimize the electric field between the tip and the sample, as judged from local contact potential difference (LCPD) measurements by the Kelvin parabola method.[10] In other words, the bias voltage was set to the maximum of the LCPD parabola. These V_s are reported in the images and correspond to the bias applied to the back of the sample plate while keeping the potential of the tip close to ground. When the surface was not irradiated by X-rays before the AFM measurements, it was not possible to compensate the potential with the maximum ±10 V voltage range delivered by the microscope controller, resulting in large absolute values of frequency shifts in the constant-height AFM images (Fig. S11 exemplifies this; background modulations due to surface charging are evident). Frequency shift vs. tip-sample distance curves were acquired on representative features on the cleaved and water-dosed surfaces as discussed in Section S6.

Ex-situ characterization

Ambient AFM images were acquired in air with an Agilent 5500 ambient AFM in intermittent contact mode with Si tips on Si cantilevers. X-ray diffraction (XRD) was used to determine the orientation of the sample using a small chip from a centimeter-sized feldspar crystal, keeping track of the mutual orientation. The chip was mounted on a Nonius KappaCCD 4-circle diffractometer run with Mo radiation. The acquisition of ten frames was sufficient to unambiguously identify the crystal orientation of the chip and of the larger feldspar crystal. For photomicrography and electron probe microanalysis (EMPA), a slab of roughly two millimeter thickness was cut from the main feldspar crystal parallel to (001) using a diamond wire saw. The slab was embedded into a stub of epoxy resin. The surface was ground and subsequently polished using diamond paste down to a grain size of 0.25 µm to obtain a smooth plane surface needed for mineral chemical analysis with EPMA. The polished rock chip had a thickness of about 500 µm and was translucent, allowing for polarization microscopy in transmitted light. Polarization microscopy was done on a Leica DM 4500 P polarization microscope with a CCD camera. For electron microscopy, the surface was carbon-coated to ensure electrical conductivity. Backscattered electron (BSE) images and mineral chemical analyses were done on a CAMECA SX Five EMPA equipped with a field emission electron source and five wavelength dispersive crystal spectrometers as well as an energy-dispersive system for elemental analysis (accelerating voltage 15 kV, beam current 20 nA). Natural mineral and synthetic oxide standards were used to calibrate quantitative mineral chemical analyses.

Computational methods (DFT)

DFT calculations were performed with the Vienna Ab-initio Simulation Package (VASP)[11,12] using the r^2SCAN-D3 metaGGA exchange-correlation functional.[13] This functional describes well the bulk structural properties; the lattice constants and angles deviate less than 0.4% from experimental values.[14] A comparison of selected values using the r^2SCAN functional[15] can be found in Section S3.

The bulk structure (Fig. 1) was optimized with a cutoff energy of 700 eV. A k-point mesh of 3 × 2 × 3 was used to integrate the Brillouin zone. The unit cell used for the calculations is the conventional cell used in the literature (dashed in Fig. 1, with the following optimized lattice parameters: a = 8.54 Å, b = 12.95 Å, c = 7.21 Å, α = 90.65°, β = 116.17°, γ = 87.61°). This is larger than the primitive unit cell (solid, Fig. 1). For studying the surfaces, the slabs were symmetric, made of 16 layers (i.e., 16 formula units of $KAlSi_3O_8$ when using the primitive unit cell in x and y), and separated by 20 Å vacuum regions, unless otherwise specified. All atoms were free to relax. The surface calculations had a cutoff energy of 400 eV and a 3 × 2 × 1 k-point mesh. Geometries were optimized using the conjugate gradient method. The structures were relaxed until residual forces on the atoms smaller than 0.01 eV/Å and an energy convergence of 10^{-6} eV were achieved.

To determine the most stable hydroxylated structures, various starting configurations with distinct OH orientations were relaxed. Several configurations were also tested for the water molecules adsorbed on the hydroxylated surface (see Section S3).

The AFM images were simulated with the Probe Particle Model,[16,17] which includes Hartree-potential electrostatics and Lennard-Jones potentials as well as the elastic properties of the tip based on the methods described in Refs. [16,17]. CuO_x and Cu tips were simulated with the following values of lateral and vertical spring constants and charges (CuO_x: $k_{x,y}$ = 161.9 N/m, k_z = 271.1 N/m, effective tip charge of −0.05 e; CO: $k_{x,y}$ = 1.7 N/m, k_z = 326.9 N/m, effective tip charge of −0.005 e; Cu: $k_{x,y}$ = 7.8 N/m, k_z = 50.7 N/m, effective tip charge of −0.05 e). Note that with values larger than 5.0 N/m, the tip's stiffness had only a minor influence on the appearance of the simulated images. The oscillation amplitude for each simulation (250−500 pm) always matched the one used in the corresponding experimental image. The best-fitting images were selected within a range of calculated tip-sample distances; distances are always referenced to the most protruding surface atom.

The chemical potential of water used in the phase diagram of Fig. S4a is defined as $\mu_{H_2O}(T,p) = \mu_{H_2O}(T,p^o) + kT\ln\left(\frac{p}{p^o}\right)$. It provides the temperature and pressure dependence given the temperature dependence of $\mu_{H_2O}(T,p^o)$ at a particular pressure p^o .[18] The reference state was chosen as the total energy $E_{H_2O}^{gas}$ of an isolated H_2O molecule in the gas phase. Assuming this reference and based on the number of water molecules N_w and formula units of bulk feldspar N_b, the surface energies of symmetric slabs of area A were calculated as $\gamma = \frac{E_{slab} - N_w E_{H_2O}^{gas} - N_b E_{bulk}^{feldspar}}{2A}$.

XPS core-level shifts were determined both in the initial and in the final-state approximations.[19,20] The shifts of the OH and H_2O components with respect to the bulk O 1s

component were calculated as (0.46 eV, 0.87 eV) and as (0.51 eV, 0.95 eV) with the two approximations, respectively.

Section S2: Further characterization of microcline feldspar

Thin-section characterization

Figure S1 displays a backscattered electron (BSE) image and a photomicrograph of a (001) oriented about 500 µm thick section of the same microcline specimen as used for the AFM experiments. Most of the sample (bright areas in the BSE image of Fig. S1a) is identified as microcline based on the composition measured by electron probe micro analysis (EPMA) as $K_{0.94}Na_{0.06}Al_{1.01}Si_{2.99}O_8$ and the typical "microcline grid" appearance in the photomicrograph of Fig. S1b (see below). In the BSE images, two types of dark grey domains are visible. Thin, nearly vertically oriented dark grey features correspond to exsolution lamellae of Na-rich alkali feldspar (albite, $NaAlSi_3O_8$), which typically form by diffusion-controlled solid-state reaction from Na-bearing K-feldspar during slow cooling from their primary crystallization temperatures.[21] The larger, irregularly shaped dark grey patches and vein-like structures are precipitates of albite, which, based on their appearance, are interpreted as having partially replaced the original K-rich feldspar in a fluid-mediated process during late-stage hydrothermal overprint. Typically, fluid-filled micro- and nano-porosity is concentrated at the interfaces between the original K-feldspar and the albite-rich precipitates.[22,23] In addition, small blebs of quartz were identified by EPMA measurements, which also appear dark grey on BSE images but are too small to be discerned in Fig. S1a. The chemical formula of the sodium-rich phases reads $Na_{0.95}K_{0.01}Ca_{0.04}Al_{1.04}Si_{2.96}O_8$, which is almost pure albite with about 4 mole % anorthite ($CaAl_2Si_2O_8$) component.

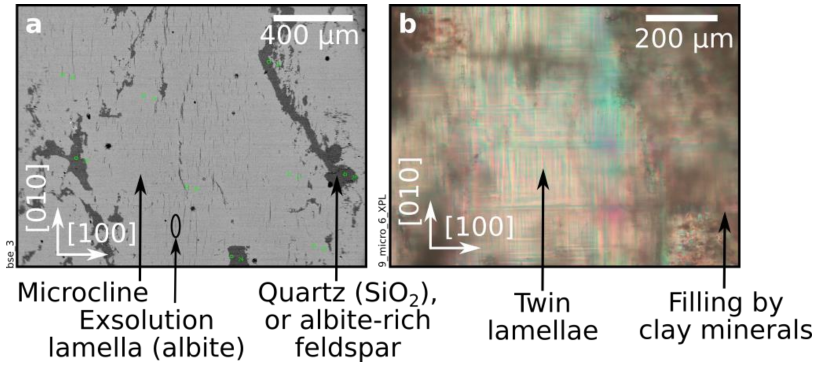

Figure S1. Characterization of a (001)-oriented section of microcline of ca. 500 µm thickness. (a) Back scattered electron (BSE) image. Bright grey: microcline, dark grey: albite and/or quartz. (b) Photomicrograph in crossed polarized transmitted light. The sub-horizontally and sub-vertically oriented band-like features are the traces of pericline (sub-horizontal) and albite (sub-vertical) twins.

The photomicrograph of Fig. S1b was acquired in cross-polarized transmitted light. Note, as the specimen is about 500 µm thick, the color shades correspond to high-order interference colors. Color contrasts indicate differences in crystal orientation. The image evidences two sets of

lamellae parallel to the traces of (010) and (100) planes. The lamellae correspond to albite twins extending parallel to (010) and pericline twins extending parallel to (100). Combined albite and pericline twining yields the characteristic “microcline grid”, a diagnostic feature of this mineral species. In addition, darker, “cloudy” areas are visible. These likely correspond to “filling” by sub-micron sized inclusions of clay minerals, a feature that is frequently observed in potassium-rich alkali feldspars and is due to late-stage hydrothermal processes.

XPS

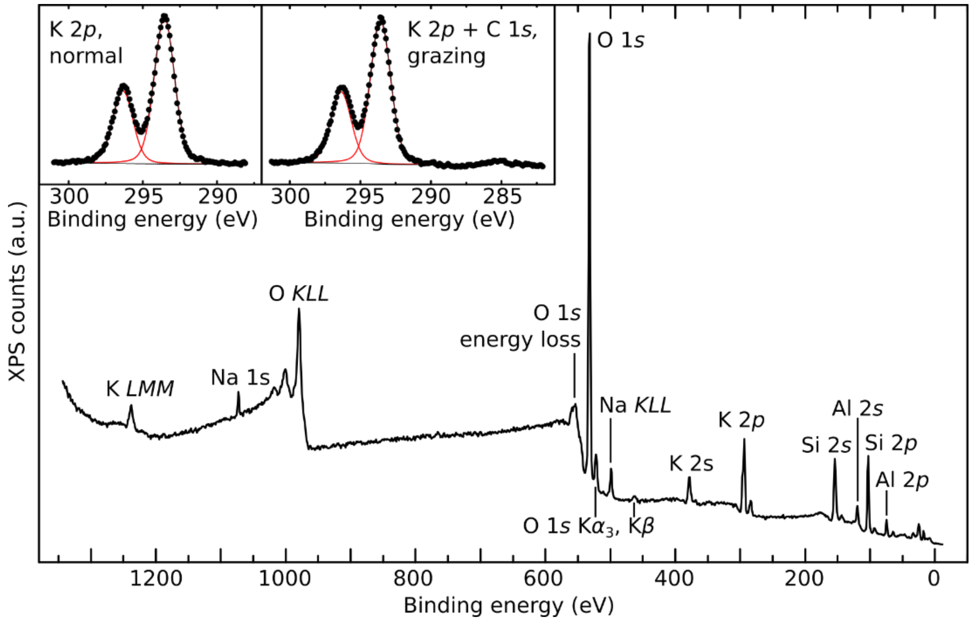

Figure S2. XPS of UHV-cleaved microcline. XPS survey (70° grazing emission) of UHV-cleaved microcline (Al Kα, 1486.61 eV, pass energy 60 eV). Inset: K 2*p* and K 2*p* + C 1*s* regions acquired in normal and grazing emission, respectively (pass energy 20 eV; Al $K\alpha_3$ satellite removed). All binding energy axes were adjusted to account for charging (see Section S1).

The XPS survey in Fig. S2 shows that the UHV-cleaved microcline features all the expected elements (K, Si, Al, O), plus a contribution of Na. As discussed above, this arises from small and sparse albite domains present in the natural mineral. As shown by the K 2*p* + C 1*s* region acquired in grazing emission, the surface is free from so-called “adventitious” carbon.

K 2*p* spectra acquired in normal and grazing emission (insets) are fit by the same contributions and look essentially identical. This is different from the K-terminated surface of muscovite mica, where surface and bulk contributions show a core-level shift of 1.22 eV. In mica, these contributions cause different peak shapes in normal- and grazing-emission spectra.[8] On the other hand, for the hydroxylated α cut of microcline, DFT predicts a core-level shift of only 0.13 eV between K 2*p* surface and bulk contributions, which cannot be resolved experimentally. This is consistent with the identical appearance of the K 2*p* spectra in normal and grazing emission. The smaller core-level shifts in microcline than in muscovite mica are likely due to the different types of surface K ions in the two cases: on microcline, the K ions are “embedded” in the surface

(see Fig. 1 in the main text); on muscovite, the K ions significantly protrude over the surface, resembling isolated adatoms.

Cleaving procedures, further ambient AFM images, optical approach in UHV

Figures S3a, b illustrate the two UHV cleaving procedures tested, which yielded consistent results. In the method shown in Fig. S3a, a metal stud is glued on the mineral's surface outside vacuum. After insertion in UHV, cleaving is induced when a wobble stick applies a tangential force to the stud while the manipulator holding the sample is gently rotated. The method shown in Fig. S3b involves a guillotine-type cleaver.[2] An appropriately shaped sample is held by a custom-made holder. The upper portion of the sample is cleaved by a blade in UHV. Compared to the first method, the guillotine has the advantage of cleaving the entire sample surface (helpful for XPS measurements), and allowing for consecutive cleavages on the same sample without breaking vacuum. On the other hand, it requires a specific sample shape in order to fit the custom-made holder.

Figure S3c shows the appearance of some portion of the cleaved surface in ambient AFM. Compared to the image shown in Fig. 2a of the main text, smaller terraces are visible. These were occasionally encountered when approaching in nc-AFM in UHV. After a lateral movement of a few hundreds of nanometers the tip usually landed in an area without steps within the scan range of the nc-AFM.

Figure S3d shows optical images of the nc-AFM tip approached to a flat area of the cleaved microcline surface. Both the tip and its reflection are visible.

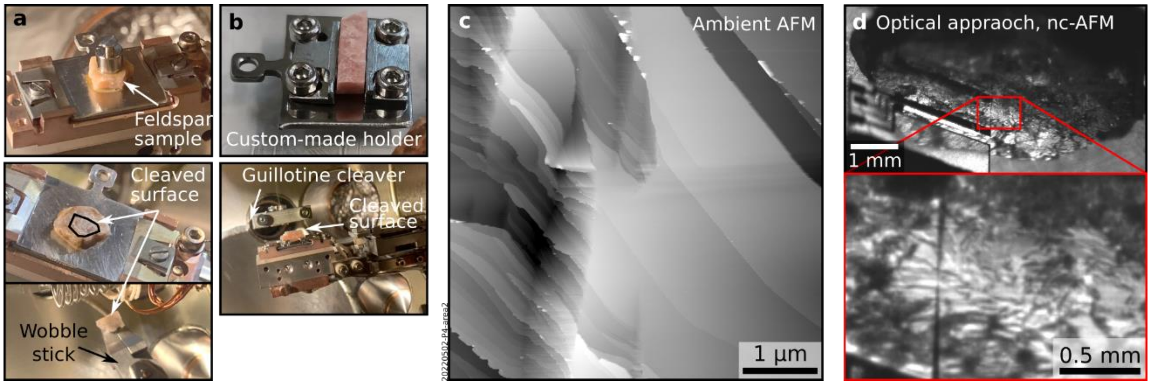

Figure S3. Cleaving microcline feldspar in UHV. (a, b) UHV cleaving was performed with either (a) a metal stud glued on the sample or (b) a guillotine-type cleaver. (c) $5 \times 5\ \mu m^2$ ambient AFM image of a region of the air-cleaved microcline surface characterized by "small" terraces. (d) Optical approach with the qPlus sensor in UHV.

Section S3: Additional computational results

Figure S4 summarizes the main outcomes of the DFT calculations performed on the α and β cuts of microcline (001). As mentioned in the main text, Al ions exclusively occupy T1 sites in the tetrahedral framework of bulk microcline[24] (see Fig. 1). Previous theoretical works have predicted that the hydroxylated surface would favor the occupation of T2 sites instead.[25] However, at room temperature, Al mobility is insufficient for them to diffuse from the bulk equilibrium sites (T1) in the tetrahedral network[24] to the less stable T2 sites. Hence, the calculations performed here maintain the occupation of the Al ions on the T1 sites.

Several calculations were tested for the hydroxylated surfaces and for the water-dosed hydroxylated surfaces. For the hydroxylated surfaces, five starting configurations with distinct OH orientations were relaxed, specifically: OH pointing vertically, and along $[100]$, $[\bar{1}00]$, $[010]$, and $[0\bar{1}0]$. The most stable configuration found for the α cut (Fig. 4b of the main text and Fig. S4c) can be obtained by initially placing the OH vertically and along [010]. It matches the one previously identified with machine-learned force fields.[26]

For the water-dosed surfaces, ten starting configurations with the H_2O molecule lying flat on the surface on different surface sites and with different orientations were relaxed. Five of them converged to the lowest-energy configuration shown in Fig. 4c and Fig. S4d, the same structure found in Ref. 25.

Additional details about the β cut

Figures S4e−g show the relaxed models for the β cut. To create the β cut (Fig. S4e), twice as many bonds need to be broken compared to the α cut. As a result, the surface energy of the β cut is significantly larger (Fig. S4h). The subsurface K ions tend to float to the surface to lower its energy when the slab thickness is insufficient. The effect is absent with large-enough slab thicknesses (as mentioned in Section S1, all calculations reported in this work have been performed with 16-layers-thick slabs, which does not induce the floating of the K ions).

Similar to the α cut, H_2O readily dissociates on the β cut (adsorption energy of −3.3 eV/H_2O). Here, two (instead of one) H_2O molecules per u.c. are needed to saturate the surface (Fig. S4f). Hence, the full hydroxylation provides a larger energy gain compared to the α cut and an overall lower surface energy (Fig. S4h), consistent with previous findings.[26] The most favored configuration corresponds to H_2O molecules donating their protons to the Al-backbonded surface O atom and their OH groups to the undercoordinated Si ions. As argued in the main text, the hydroxylated β cut will not be observed experimentally even though it is theoretically preferred at 0 K. A partial coverage (1 H_2O/u.c.) was also tried, but it was unfavorable compared to the fully hydroxylated surface at all values of the water chemical potential where hydroxylation occurs.

a

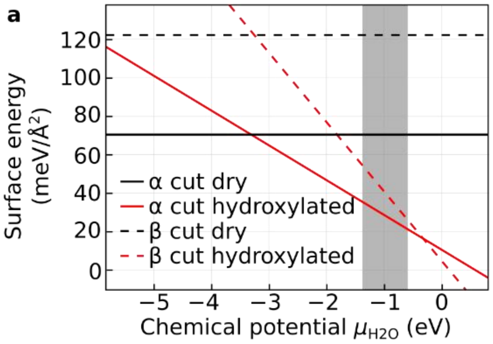

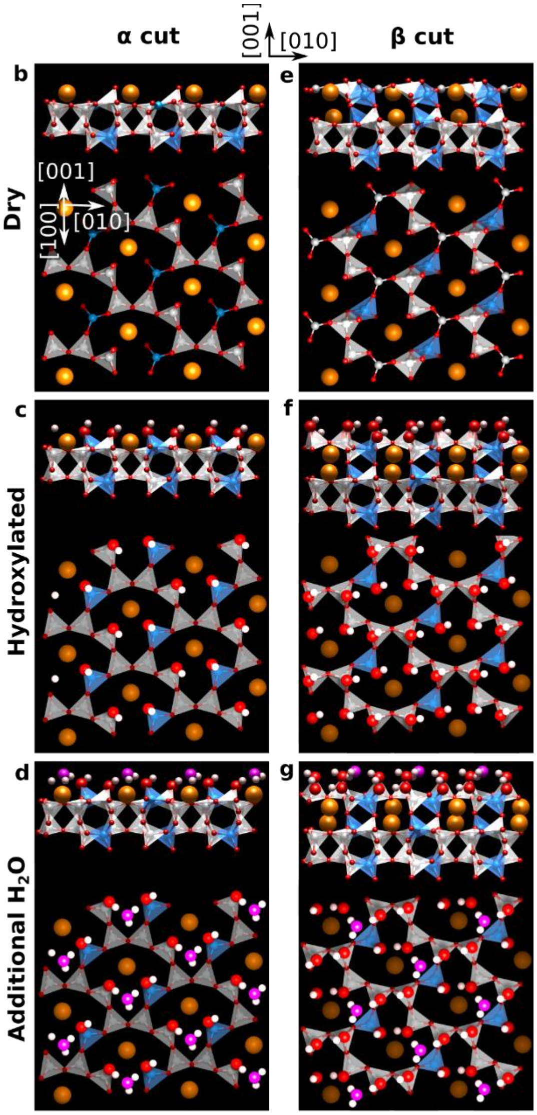

h

	Surface termination	E_{surf} (meV/Å²)		E_{ads}/H_2O (eV)	
α	Dry	73	67	-	-
	Hydroxylated ($1\ H_2O$/u.c.)	13	8	−3.3	−3.2
	1 additional H_2O ($2\ H_2O$/u.c.)	-	-	−0.8	−0.7
β	Dry	123	117	-	-
	Hydroxylated ($2\ H_2O$/u.c.)	3	1	−3.3	−3.3
	1 additional H_2O ($3\ H_2O$/u.c.)	-	-	−0.9	−0.8

i

Bulk values		Experim. (Ref. 14)	DFT (r^2SCAN-D3)	DFT (r^2SCAN)
Lattice constants (Å)	*a*	8.57	8.54	8.58
	b	12.94	12.95	13.00
	c	7.21	7.21	7.25
Angles (°)	*α*	90.53	90.65	90.73
	β	115.97	116.17	116.08
	γ	87.97	87.61	87.57
Volume (Å³)		718.23	715.32	725.47

Figure S4. **Additional computational results on the (001) microcline surface.** (a) Phase diagram as a function of the chemical potential of water (see definition in Section 1) obtained with the r^2SCAN-D3 functional. The gray region identifies the pressure range between 1×10^{-11} mbar and 6 mbar at 300 K. (b–d) Surface structures of relaxed α cuts with (b) no water adsorbed, (c) 1 dissociated H_2O/u.c., and (d) 1 H_2O molecule/u.c. on top of the hydroxylated surface (reproduced from the main text for reference). (e–g) Relaxed β cuts with (e) no water adsorbed, (f) 2 dissociated H_2O/u.c., and (g) 1 H_2O/u.c. on top of the hydroxylated surface. (h) Surface energies for μ_{H2O} = 0 and water adsorption energies on the different terminations obtained with r^2SCAN-D3 (black) and the r^2SCAN (green) functionals. For the structures containing molecular H_2O, differential adsorption energies are given (i.e., the energy of adding a water molecule to the hydroxylated surface). (i) DFT-optimized bulk constants (lattice vectors and angles) and equilibrium volumes obtained with the r^2SCAN-D3 (black) and r^2SCAN (green) functionals, compared to experiment.[14]

For completeness, it was also investigated how additional H_2O adsorbs on the hydroxylated β cut – even though, as argued before, this cut was not found to result from cleaving. The most stable configuration is shown in Fig. S4g (adsorption energy of −0.9 eV).

The phase diagram in Fig. S4a summarizes the results obtained for the dry and hydroxylated α and β cuts. It plots their surface energies as a function of the water chemical potential μ_{H_2O} as defined in the Section S1. The dry α cut is more stable than the β cut at all conditions because the β cut requires breaking more bonds. If there is enough water available, both cuts will become hydroxylated. Under UHV-compatible conditions and in the ambient atmosphere, the hydroxylated α cut is more stable. The hydroxylated β cut may become stable under liquid conditions.

Additional details about the α cut

To cleave microcline (001) at the α plane and retain a polarity-compensated surface, 50% of the O atoms lying at the same height as the K ions must be removed. These O atoms are not equivalent, as they are bound to either Si or Al. Hence, different ways exist to cleave the surface. Figure S5 shows side views of the relaxed DFT models obtained by cleaving the slab in three ways and the corresponding surface energies. The lowest-energy structure (used throughout the main text and consistent with the bulk structure used in previous studies[25]) is obtained by exclusively cleaving Al-O bonds and retaining Si-O bonds at the surface (Fig. S5a). The surface energy is significantly larger when the surface is obtained by cleaving Si-O bonds and maintaining Al-O bonds (Fig. S5c). Figure S5b (intermediate surface energy) was obtained by cleaving both Al-O and Si-O bonds. These results are as expected when considering the higher formal charge of the Si atoms (4+) compared with Al (3+).

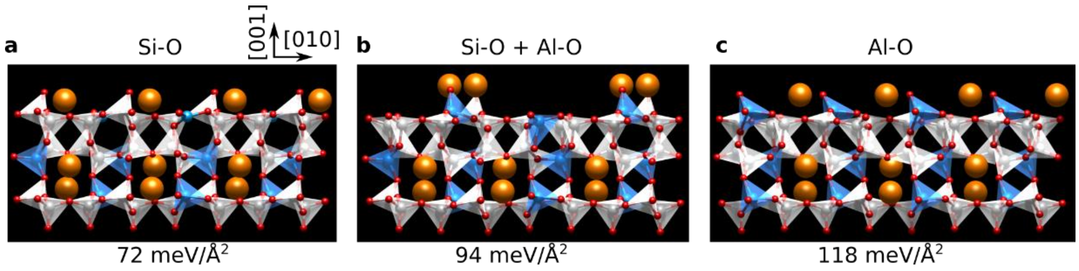

Figure S5. Relaxed DFT models and surface energies of the α cut of microcline (001) obtained by cleaving different bonds. (a) Al-O bonds are broken, Si-O bonds remain on the surface. (b) Both Si-O and Al-O bonds are broken. (c) Si-O bonds are broken, Al-O bonds remain.

Performance of r^2SCAN and r^2SCAN-D3 functionals compared

As shown in Fig. S4i, the optimized r^2SCAN and r^2SCAN-D3 lattice parameters are in excellent agreement with the experimental values[14] (maximal deviation of ~0.5% and ~0.4%, respectively). The r^2SCAN-D3 functional results in only slightly smaller lattice parameters a and c, leading to a minor underestimation of the optimized equilibrium volume compared to the experimental one. The Grimme D3 corrections only slightly increase the surface energies (<6 meV/Å^2) and increase the adsorption energies of H_2O (<0.1 eV/H_2O) on the different terminations, see Figure S4h.

Section S4: Considerations about symmetry

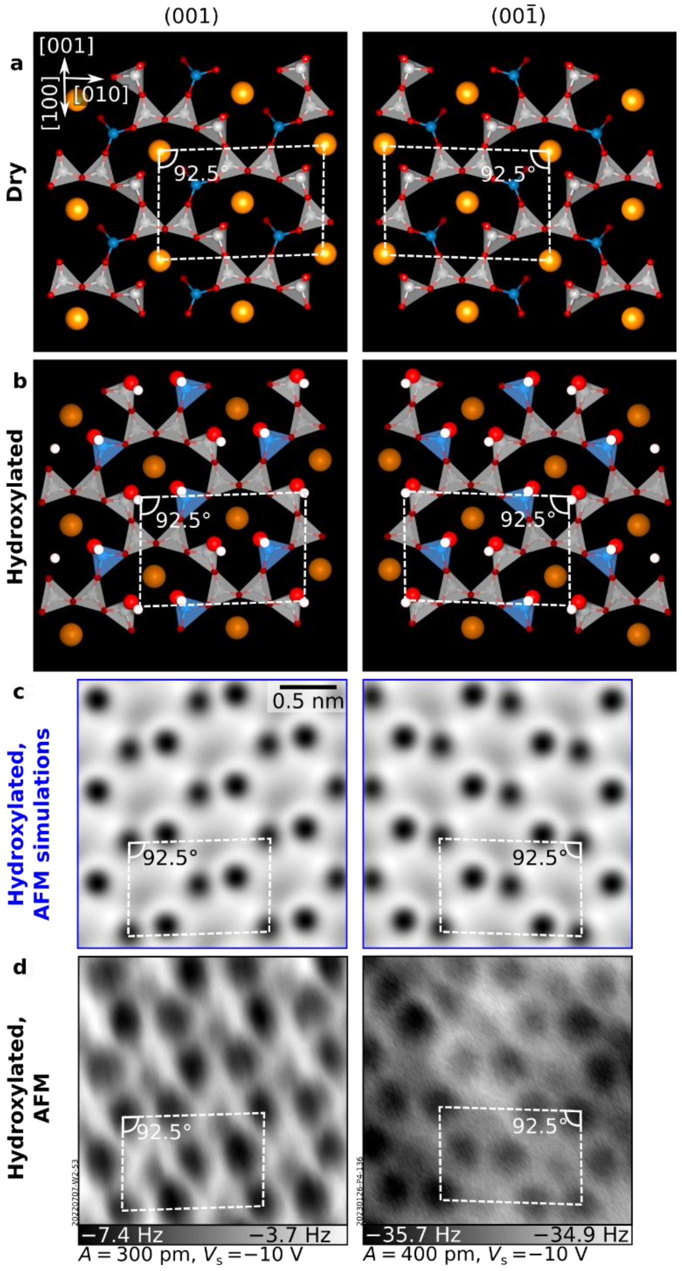

Figure S6. Considerations about symmetry. (a) Top views of the (001) and (00$\bar{1}$) terminations of microcline. The structures are mirror-symmetric with respect to the vertical axis. (b, c) Top views and 2.5 × 2.5 nm^2 AFM simulations of the hydroxylated (001) and (00$\bar{1}$) cuts of microcline. (d) AFM images of two hydroxylated microcline samples imaged with similar tips. The images are mirror symmetric.

As mentioned in the main text, microcline has a centrosymmetric crystal structure. This means that the (001) and (00$\bar{1}$) facets, while equal in energy, are not equivalent (Fig. S6a). They differ in the positions of the Al ions relative to the tetrahedral framework (related by mirror symmetry).

The same considerations hold for the hydroxylated surface (Fig. S6b). Because the AFM contrast is dominated by the OH groups attached to the Al ions, images simulated on the (001) vs.

$(00\bar{1})$ terminations are also mirror symmetric (Fig. S6c). Mirror-symmetric facets have been observed experimentally on differently oriented samples (Fig. S6d). The main text presents images acquired mostly on one type of termination, referred to as (001).

Section S5: Additional experimental and simulated AFM images

Figure S7 illustrates the sensitivity of the AFM contrast to tip terminations. Figure S7a was acquired with a Cu tip (albeit less sharp than the Cu tip used to image the surface as in Fig. 2c in the main text). Figures S7b, c were acquired with tips modified by the interaction with some point defects present at the microcline surface, likely adsorbates. These so-called ‘feldspar-modified’ tips produce a similar honeycomb pattern as that seen with the sharpest Cu- or CuO_x-terminated tips (see main text). Instead, the slightly blunt Cu tip shows a hexagonal pattern with a poor signal-to-noise ratio (approaching any closer induces inadvertent tip changes). Figures S7d, e show the same area of a $(00\bar{1})$-oriented sample imaged with two different tips. Note that the images are mirror-symmetric compared to the ones in the other panels, in which the sample exposes its (001) facet (see Section S4 above for details about symmetry considerations). Figure S7d was acquired with a (slightly blunt) Cu-prepared tip. Figure S7e shows an image after interaction with a point defect at the surface, which produces the most common (honeycomb) contrast observed across several samples. The images were aligned using the surface defects as reference. This Cu tip provides a repulsive contrast at the Al-OH (white triangle) and Si-OH (black triangle) positions.

Figures S8a, b highlight the sensitivity of the imaging contrast to the tip-sample separation. In both, the experimental and simulated AFM images, the honeycomb pattern appears more pronounced when approaching closer.

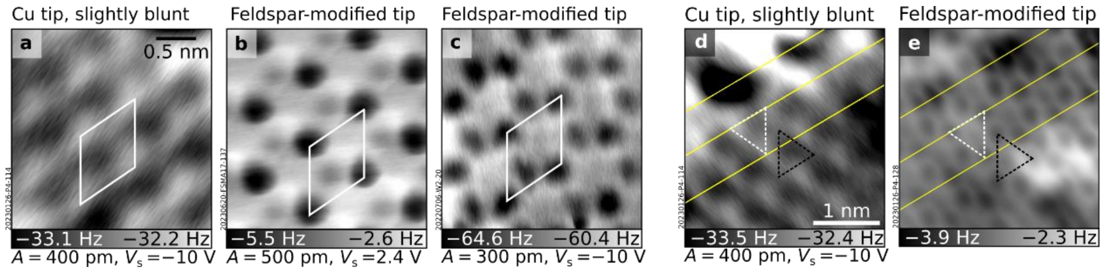

Figure S7. Imaging with different tip terminations. (a−c) 2.5 × 2.5 nm^2 AFM images of UHV-cleaved (001) microcline surfaces measured with the tips specified. The image in panel (a) has been mirrored for displaying purposes – it was acquired on the mirror-symmetric $(00\bar{1})$ orientation, see Section S4. It was acquired with a Cu tip less sharp than the Cu tip used to acquire the image in Fig. 2c of the main text. (d, e) 3 × 3 nm^2 images AFM images acquired on the same area of a $(00\bar{1})$-oriented sample (yellow lines are meant to guide the eye). White and black triangles identify Al-OH and Si-OH, respectively.

Figures S8c, d compare experimental and simulated images of the water-dosed microcline surface acquired with a Cu-terminated tip. The water features appear dark (attractive) at all tip-sample distances explored. On the other hand, Figs. 5c, d of the main text show that CO-terminated

tips produce a repulsive (bright) contrast on the water species. Such differences are expected. According to the model in Fig. 4c, the adsorbed H_2O molecules are arranged with an O atom pointing up. Such an O atom should be imaged in the repulsive regime with O-terminated tips, and in the attractive regime with Cu-terminated tips.

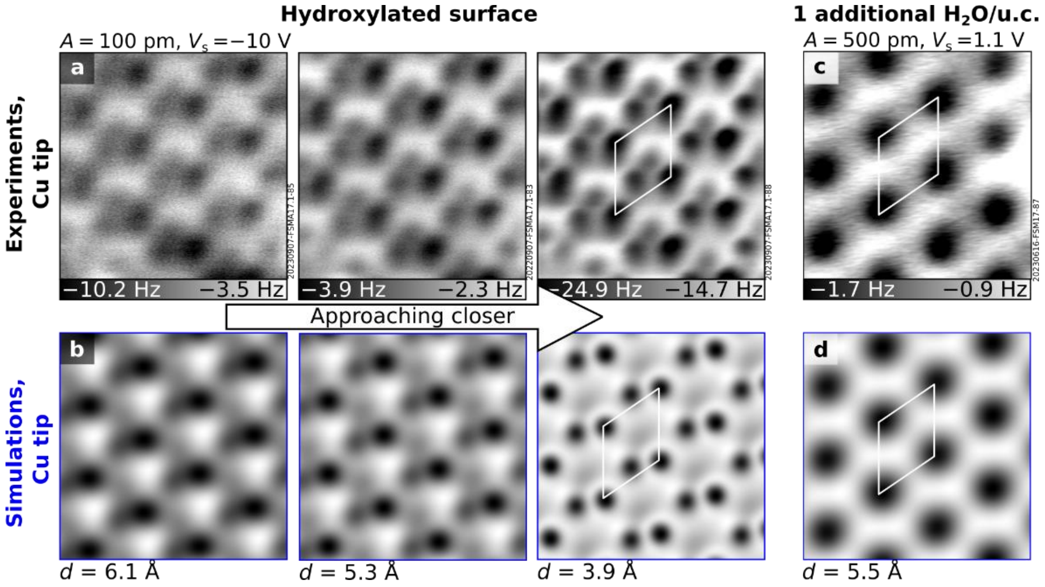

Figure S8. Experimental and simulated AFM images with Cu tips. 2.7 × 2.7 nm^2 experimental (top) and simulated (bottom) AFM images acquired with a Cu tip of the (a, b) hydroxylated, and (c, d) water-dosed microcline (001) α cut. White rhombi identify the primitive unit cells.

Section S6: Δ*f*-*z* curves

Curves of frequency shifts vs. tip-sample distance were acquired on representative features on the cleaved and water-dosed microcline (001) surface (Figs. S9c, f). They are marked with the same color coding in the experimental images of Figs. S9b, e and in the proposed DFT models in Figs. S9a, d.

Before each acquisition, the tip was positioned on the chosen feature and retracted by 1 nm from the acquisition height of the images shown in Figs. S9b, e. The two sets of data were acquired with different tips (oxygen-terminated in the case of the hydroxylated surface and modified by the interaction with water on the water-dosed surface), preventing quantitative comparisons. Nonetheless, they can provide rough indications of the vertical separations between the different surface species. A more quantitative investigation of force-distance interactions would demand the acquisition of numerous curves with different, carefully prepared tips and is beyond the scope of this work.

On the cleaved surface (Fig. S9b), curves were acquired on various features discernible at the surface. Based on the assignments in the main text, these correspond to Si-OH (black), Al-OH (light blue), K (orange), and the background between the protruding species (green). The Si-OH curve displays a clear minimum. A minimum is also present for the Al-OH. The curves on the two

hydroxyls have their minimum almost at the same z position, in reasonable agreement with the DFT model that predicts a vertical separation between the two hydroxyls of ≈40 pm (to be precise, the vertical separation between the H atoms in the hydroxyls is 50 pm; the one between the O atoms is 26 pm). The Δf separation of the two minima is ≈7 Hz, indicating a different interaction strength of the two types of hydroxyls with the O-terminated tip.[27]

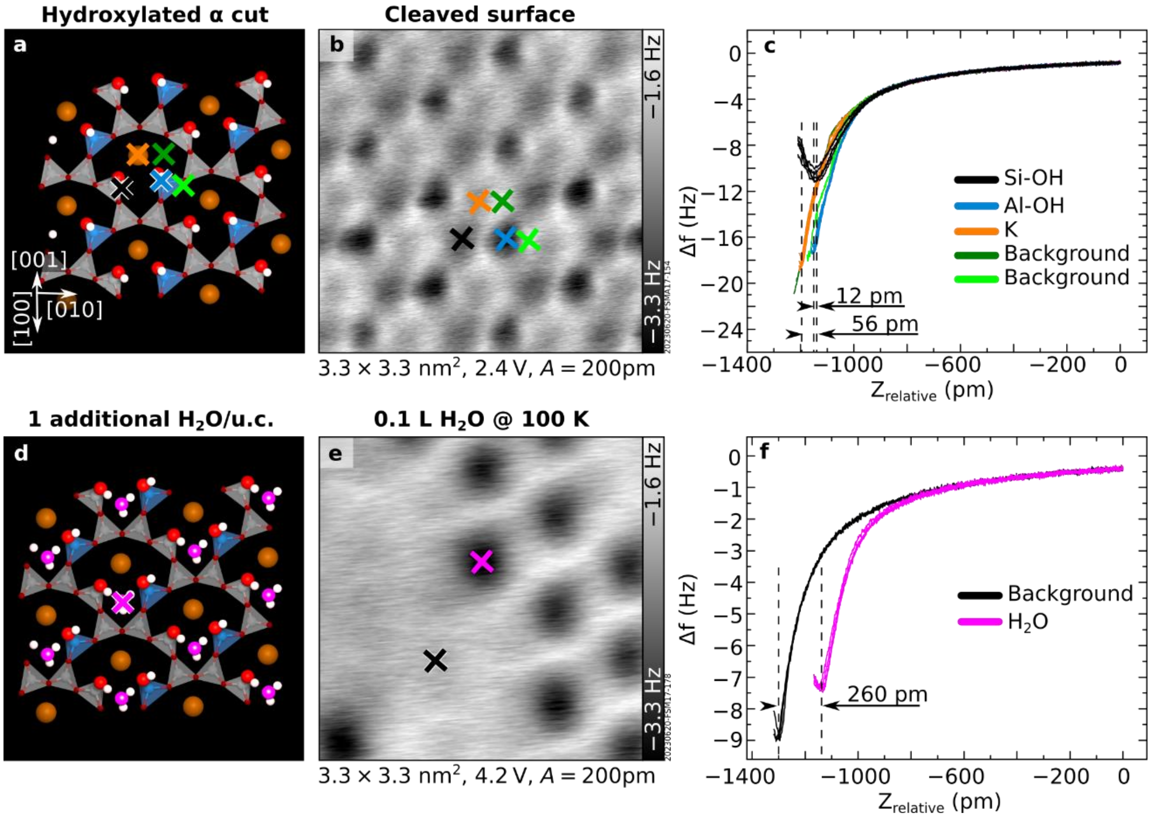

Figure S9. Force-distance curves. (a) DFT-relaxed model of the hydroxylated α cut. (c) Δf-z curves acquired on the spots marked in panel (b) with a tip modified by the interaction with a water species. (d) DFT-relaxed model of water of the hydroxylated α cut. (f) Δf-z curves acquired on the spots marked in panel (e) with an oxygen-terminated tip.

The minimum of the curve acquired on top of the K ion is shifted by roughly 60 pm in z, in reasonable agreement with the calculated vertical distance of ≈90 pm between the surface K ions and the hydroxyls. The curves acquired on the background display the same long-range interactions as the other curves; they do not reach a minimum before −20 Hz.

On the water-dosed surface (Fig. S9d), curves were acquired on several spots on the background (black) and on the water species protruding over the surface (pink). Minima are observed in both cases and are separated by ≈260 pm in height. This indicates that the water species significantly protrude over the surface, in accordance with the DFT models that predict vertical separations of 125 pm and 235 pm between the protruding H_2O and the surface hydroxyls and K ions, respectively.

Section S7: Further arguments for the ready hydroxylation of the as-cleaved surface

Figures S10a, b compare AFM images of the microcline (001) surface after cleaving and after dosing 0.2 Langmuir (see Section S1 for the definition of Langmuir) H_2O at 100 K, followed by warm-up to 300 K. Because of the high adsorption energies of the hydroxyls predicted by DFT, one expects an initially completely 'dry' surface to readily hydroxylate when exposed to water at low temperatures, and also to keep the hydroxyls when the sample is warmed to 300 K, where additional molecular water desorbs. The similar appearance of the surface after this treatment compared to the as-cleaved surface suggests that hydroxyls were already present after the cleaving.

Figure S10c displays the partial pressure of water during cleaving, as measured with a quadrupole mass-spectrometer in the UHV chamber. A partial pressure spike up to 2×10^{-7} mbar is visible. The total integrated area yields a dose of ≈ 5 L, which is much larger than the nominal dose of 0.32 L required to fully hydroxylate the sample. Moreover, the water vapor pressure measured by the mass spectrometer will be smaller than the one close to the sample surface, where the water was released.

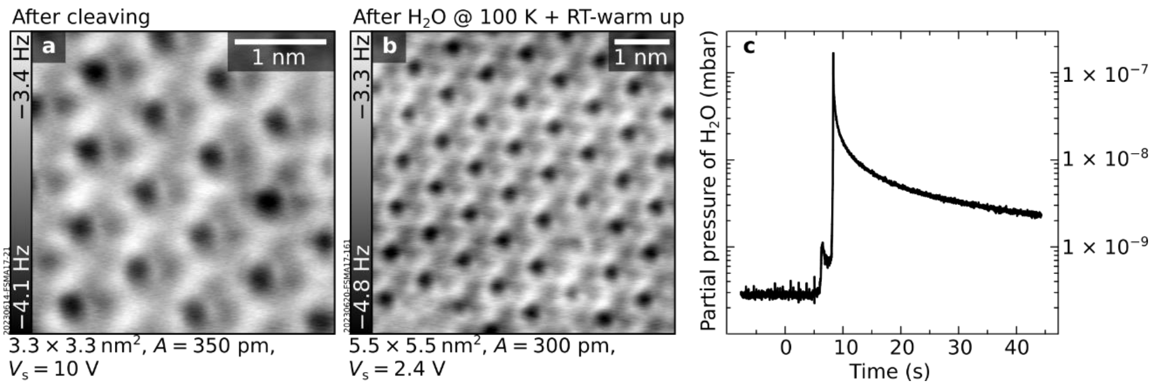

Figure S10. Ready hydroxylation of microcline in UHV. AFM images of microcline (001), taken at 4 K, (a) after UHV cleaving and (b) after dosing 0.2 L H_2O at 100 K followed by warming up to room temperature for 20 min. (c) Partial pressure of mass 18 measured by a mass-spectrometer while cleaving a microcline crystal in UHV.

Section S8: Imaging in the presence of surface charges

As for other insulators, the microcline (001) surface displays significant charging after cleaving in UHV. If the charge is not compensated by irradiating with X-rays or by applying a sufficiently large bias voltage (see Section S1), imaging the surface is challenging. Electrostatic interactions between the surface and the tip dominate the force interactions at large distances, making it hard to judge when the tip is approached to the sample. Once approached, severe background modulations dominate the contrast, complicating the assessment of the surface's tilt and its appropriate correction. Figure S11 shows an example of a (mildly) charged surface. Bright and dark modulations overlap with the microcline lattice. In more severe cases, the background modulations are so strong that they hinder any atomic resolution.

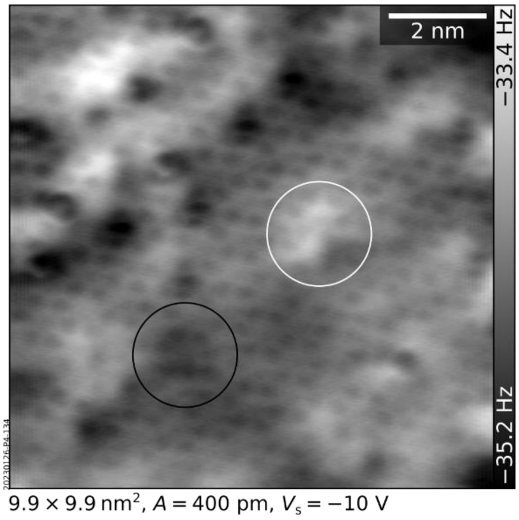

Figure S11. Appearance of a charged microcline surface in AFM. Brighter and darker areas (white and black circles) are due to trapped charges.

Supplementary References

(1) Stöger, B.; Hieckel, M.; Mittendorfer, F.; Wang, Z.; Schmid, M.; Parkinson, G. S.; Fobes, D.; Peng, J.; Ortmann, J. E.; Limbeck, A.; Mao, Z.; Redinger, J.; Diebold, U. Point Defects at Cleaved $Sr_{n+1}Ru_nO_{3n+1}$(001) Surfaces. *Phys. Rev. B* **2014**, *90*, 165438. https://doi.org/10.1103/PhysRevB.90.165438.

(2) Sokolović, I.; Schmid, M.; Diebold, U.; Setvin, M. Incipient Ferroelectricity: A Route towards Bulk-Terminated $SrTiO_3$. *Phys. Rev. Mater.* **2019**, *3* (3), 034407. https://doi.org/10.1103/PhysRevMaterials.3.034407.

(3) Malins, A. E. R.; Poolton, N. R. J.; Quinn, F. M.; Johnseir, O.; Denby, P. M. Luminescence Excitation Characteristics of Ca, Na and K-Aluminosilicates (Feldspars) in the Stimulation Range 5-40 eV: Determination of the Band-Gap Energies. *J. Phys. D. Appl. Phys.* **2004**, *37* (10), 1439–1450. https://doi.org/10.1088/0022-3727/37/10/005.

(4) Kyono, A.; Kimata, M.; Hatta, T. Aluminum Position in Rb-Feldspar as Determined by X-Ray Photoelectron Spectroscopy. *Naturwissenschaften* **2003**, *90* (9), 414–418. https://doi.org/10.1007/s00114-003-0453-0.

(5) Huber, F.; Giessibl, F. J. Low Noise Current Preamplifier for QPlus Sensor Deflection Signal Detection in Atomic Force Microscopy at Room and Low Temperatures. *Rev. Sci. Instrum.* **2017**, *88* (7), 073702. https://doi.org/10.1063/1.4993737.

(6) Setvín, M.; Javorský, J.; Turčinková, D.; Matolínová, I.; Sobotík, P.; Kocán, P.; Ošťádal, I. Ultrasharp Tungsten Tips—Characterization and Nondestructive Cleaning. *Ultramicroscopy* **2012**, *113*, 152–157. https://doi.org/10.1016/J.ULTRAMIC.2011.10.005.

(7) Schulze Lammers, B.; Yesilpinar, D.; Timmer, A.; Hu, Z.; Ji, W.; Amirjalayer, S.; Fuchs, H.; Mönig, H. Benchmarking Atomically Defined AFM Tips for Chemical-Selective Imaging. *Nanoscale* **2021**, *13* (32), 13617–13623. https://doi.org/10.1039/d1nr04080d.

(8) Franceschi, G.; Kocán, P.; Conti, A.; Brandstetter, S.; Balajka, J.; Sokolović, I.; Valtiner, M.; Mittendorfer, F.; Schmid, M.; Setvín, M.; Diebold, U. Resolving the Intrinsic Short-Range Ordering of K^+ Ions on Cleaved Muscovite Mica. *Nat. Commun.* **2023**, *14* (1), 208. https://doi.org/10.1038/s41467-023-35872-y.

(9) Ostendorf, F.; Schmitz, C.; Hirth, S.; Kühnle, A.; Kolodziej, J. J.; Reichling, M. How Flat Is an Air-Cleaved Mica Surface? *Nanotechnology* **2008**, *19* (30), 305705. https://doi.org/10.1088/0957-4484/19/30/305705.

(10) Sadewasser, S.; Glatzel, T. *Kelvin Probe Force Microscopy*; Sadewasser, S., Glatzel, T., Eds.; Springer Series in Surface Sciences; Springer International Publishing: Cham, 2012; Vol. 48. https://doi.org/10.1007/978-3-642-22566-6.

(11) Kresse, G.; Hafner, J. Ab Initio Molecular Dynamics for Liquid Metals. *Phys. Rev. B* **1993**, *47* (1), 558–561. https://doi.org/10.1103/PhysRevB.47.558.

(12) Kresse, G.; Furthmüller, J. Efficiency of Ab-Initio Total Energy Calculations for Metals and Semiconductors Using a Plane-Wave Basis Set. *Comput. Mater. Sci.* **1996**, *6* (1), 15–50. https://doi.org/10.1016/0927-0256(96)00008-0.

(13) Ehlert, S.; Huniar, U.; Ning, J.; Furness, J. W.; Sun, J.; Kaplan, A. D.; Perdew, J. P.; Brandenburg, J. G. R^2SCAN-D4: Dispersion Corrected Meta-Generalized Gradient Approximation for General Chemical Applications. *J. Chem. Phys.* **2021**, *154* (6), 61101. https://doi.org/10.1063/5.0041008.

(14) Allan, D. R.; Angel, R. J. A High-Pressure Structural Study of Microcline ($KAlSi_3O_8$) to 7 GPa. *Eur. J. Mineral.* **1997**, *9* (2), 263–276. https://doi.org/10.1127/ejm/9/2/0263.

(15) Furness, J. W.; Kaplan, A. D.; Ning, J.; Perdew, J. P.; Sun, J. Accurate and Numerically Efficient r^2SCAN Meta-Generalized Gradient Approximation. *J. Phys. Chem. Lett.* **2020**, *11* (19), 8208–8215. https://doi.org/10.1021/acs.jpclett.0c02405.

(16) Hapala, P.; Kichin, G.; Wagner, C.; Tautz, F. S.; Temirov, R.; Jelínek, P. Mechanism of High-Resolution STM/AFM Imaging with Functionalized Tips. *Phys. Rev. B* **2014**, *90* (8), 85421.

https://doi.org/10.1103/PhysRevB.90.085421.
(17) Hapala, P.; Temirov, R.; Tautz, F. S.; Jelínek, P. Origin of High-Resolution IETS-STM Images of Organic Molecules with Functionalized Tips. *Phys. Rev. Lett.* **2014**, *113* (22), 226101. https://doi.org/10.1103/PhysRevLett.113.226101.
(18) Reuter, K.; Scheffler, M. Composition, Structure, and Stability of (Formula Presented) as a Function of Oxygen Pressure. *Phys. Rev. B* **2002**, *65* (3), 1–11. https://doi.org/10.1103/PhysRevB.65.035406.
(19) Köhler, L.; Kresse, G. Density Functional Study of CO on Rh(111). *Phys. Rev. B* **2004**, *70* (16), 165405. https://doi.org/10.1103/PhysRevB.70.165405.
(20) Lizzit, S.; Baraldi, A.; Groso, A.; Reuter, K.; Ganduglia-Pirovano, M. V.; Stampfl, C.; Scheffler, M.; Stichler, M.; Keller, C.; Wurth, W.; Menzel, D. Surface Core-Level Shifts of Clean and Oxygen-Covered Ru(0001). *Phys. Rev. B* **2001**, *63* (20), 205419. https://doi.org/10.1103/PhysRevB.63.205419.
(21) Abart, R.; Petrishcheva, E.; Wirth, R.; Rhede, D. Exsolution by Spinodal Decomposition II: Perthite Formation during Slow Cooling of Anatexites from Ngoronghoro, Tanzania. *Am. J. Sci.* **2009**, *309* (6), 450–475. https://doi.org/10.2475/06.2009.02.
(22) Parsons, I.; Gerald, J. D. F.; Lee, M. R. Review. Routine Characterization and Interpretation of Complex Alkali Feldspar Intergrowths. *Am. Mineral.* **2015**, *100* (5–6), 1277–1303. https://doi.org/10.2138/am-2015-5094.
(23) Fitz Gerald, J. D.; Parsons, I.; Cayzer, N. Nanotunnels and Pull-Aparts: Defects of Exsolution Lamellae in Alkali Feldspars. *Am. Mineral.* **2006**, *91* (5–6), 772–783. https://doi.org/10.2138/am.2006.2029.
(24) Kroll, H.; Ribbe, P. H. Determining (Al,Si) Distribution and Strain in Alkali Feldspars Using Lattice Parameters and Diffraction-Peak Positions: A Review. *Am. Mineral.* **1987**, *72* (5–6), 491–506.
(25) Pedevilla, P.; Cox, S. J.; Slater, B.; Michaelides, A. Can Ice-Like Structures Form on Non-Ice-Like Substrates? The Example of the K-Feldspar Microcline. *J. Phys. Chem. C* **2016**, *120* (12), 6704–6713. https://doi.org/10.1021/ACS.JPCC.6B01155.
(26) Piaggi, P.; Selloni, A.; Panagiotopoulos, A. Z.; Car, R.; Debenedetti, P. G. A First-Principles Machine-Learning Force Field for Hetero- Geneous Ice Nucleation on Microcline Feldspar. *Faraday Discuss.* **2023**. https://doi.org/10.1039/D3FD00100H.
(27) Wagner, M.; Meyer, B.; Setvin, M.; Schmid, M.; Diebold, U. Direct Assessment of the Acidity of Individual Surface Hydroxyls. *Nature* **2021**, *592* (7856), 722–725. https://doi.org/10.1038/s41586-021-03432-3.
